# Hip Fracture in a Patient with Primary Hyperparathyroidism: Medical and Surgical Lessons

**DOI:** 10.7759/cureus.2104

**Published:** 2018-01-23

**Authors:** MN Baig, Cathal Mac Dhaibheid, Fintan J Shannon

**Affiliations:** 1 Trauma & Orthopaedics, Galway University Hospital

**Keywords:** hyperparathyroidism, osteitis fibrosa cystica

## Abstract

Hyperparathyroidism is a condition which can be primary, secondary or tertiary and is characterized by increased calcium levels, low phosphate levels, and elevated parathyroid hormone (PTH) levels. Primary hyperthyroidism can cause severe bone resorption leading to bone pains and pathological fracture. We present the case of a patient with severe primary hyperparathyroidism with an atraumatic fracture at the neck of the femur and multiple medical comorbidities presenting a surgical challenge. While primary hyperparathyroidism is rare, it should be considered during differential diagnoses.

## Introduction

Parathyroid hormone (PTH) is released by the parathyroid glands located behind the thyroid gland. It is the main hormone involved in calcium hemostasis [1].PTH increases plasma calcium levels by liberating calcium from the bone, increasing intestinal absorption of calcium, and reducing urinary excretion of calcium. PTH also decreases phosphate levels via inhibiting reabsorption in the kidneys.

Hyperparathyroidism is the overproduction of PTH, a condition that may be due to primary, secondary or tertiary causes. Primary hyperparathyroidism is caused by hypersecretion of PTH by parathyroid hyperplasia or adenoma. Biochemically, hyperparathyroidism raises PTH levels and serum calcium levels and decreases serum phosphate levels. It is usually diagnosed as an incidental finding on routine biochemical tests. Clinically, most primary hyperparathyroidism patients are asymptomatic, but some nonspecific symptoms secondary to high calcium levels are notable; these include fatigue, joint aches, weakness, mild depression, and difficulty concentrating [2].

To diagnose primary hyperparathyroidism, we must rule out other causes of parathyroidism and correlate the condition with biochemical results. Most cases are reported in women and peak in the seventh decade of life [3]. Severe bone disease with severe primary hyperparathyroidism is called osteitis fibrosa. Very rarely they present with hip fractures, and it is a challenge to optimize them medically and treat them surgically [4]. We present the case of a 77-year-old woman presenting with hip fracture and a history of primary parathyroidism.

## Case presentation

A 77-year-old woman was referred to the emergency department by her general physician with concerns of a three-day history of progressively worsening right hip pain and an inability to fully bear weight on the affected side. The patient reported no history of trauma, fall, or any other injury. Of note, the patient suffered a previous fracture of the left patella the previous year. Her past medical history was significant for progressive Stage V chronic kidney disease; hyperparathyroidism (diagnosed in 2003 and previous subtotal parathyroidectomy was in 2004), and thrombotic microangiopathy.

On examination, we noted no swelling, ecchymosis, or deformity in her right hip. There was no external shortening or rotation of the leg. The main finding on physical examination was her inability to bear weight and a restricted range of movement in her right hip.

Her estimated glomerular filtration rate was 7 mL/minute, her PTH level was a remarkable 3842.0 ng/L, and her serum calcium was 2.8 mmol/L. All these levels were much higher than the reference ranges.

**Figure 1:**
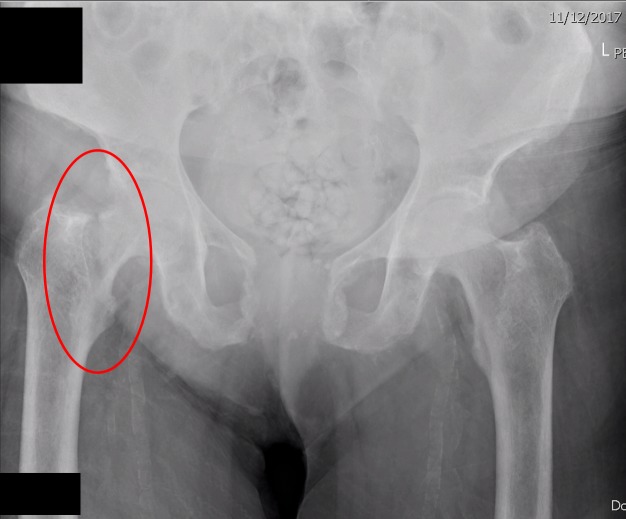
X-ray of the pelvis - basicervical fracture X-ray of the antero-posterior pelvis revealing abnormal bone texture in the right proximal femur with a cortical breach superiorly in the femoral neck.

An x-ray of the anteroposterior pelvis revealed abnormal bone texture in the right proximal femur with a cortical breach superiorly in the femoral neck (Figure 1). Chronic erosive symphysitis of the pubic symphysis had deteriorated relative to the previous x-rays.

**Figure 2:**
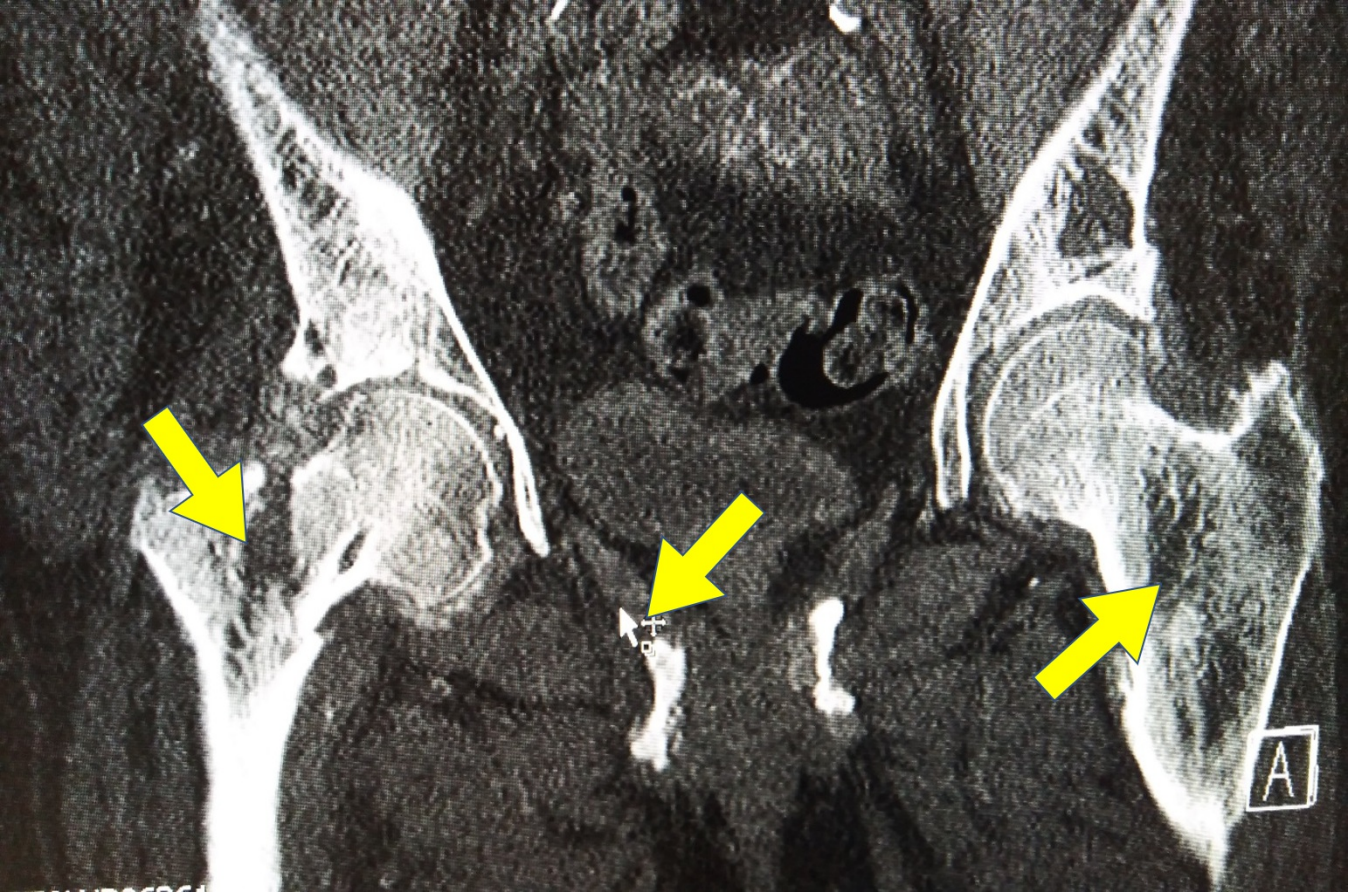
Computed tomography (CT) scan of the pelvis - axial view The fracture is visible with extensive bone resorption not only in the right femur neck but also in the ischium and contralateral femur.

The x-rays were followed by a computed tomography (CT) scan of the pelvis to provide further information for an optimal management plan. In the CT scan, the fracture is visible with extensive bone resorption not only in the right femur neck but also in the ischium and contralateral femur (Figure 2).

**Figure 3:**
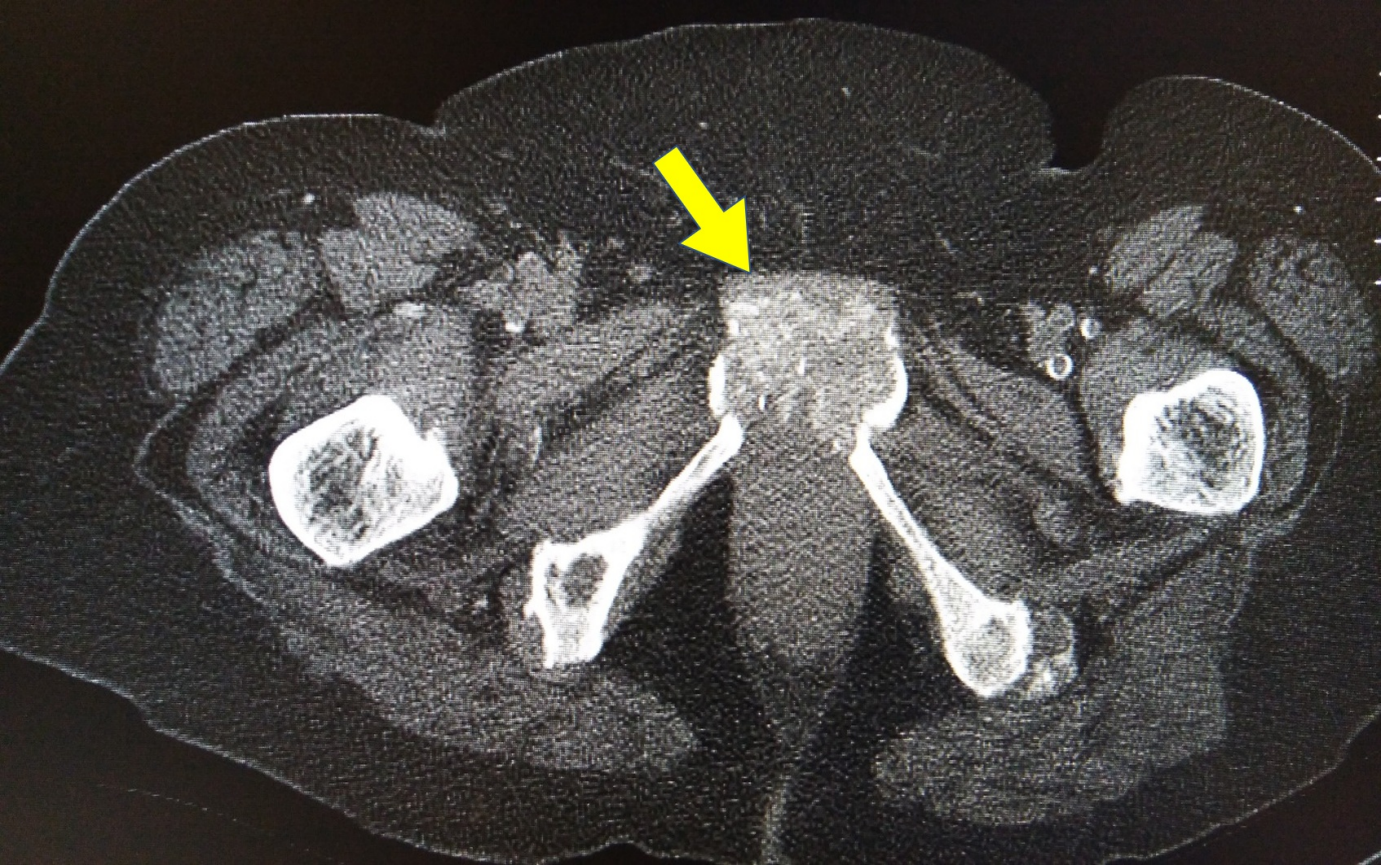
Computed tomography (CT) scan of the pelvis - coronal view Chronic symphysitis of pubic symphysis.

**Figure 4:**
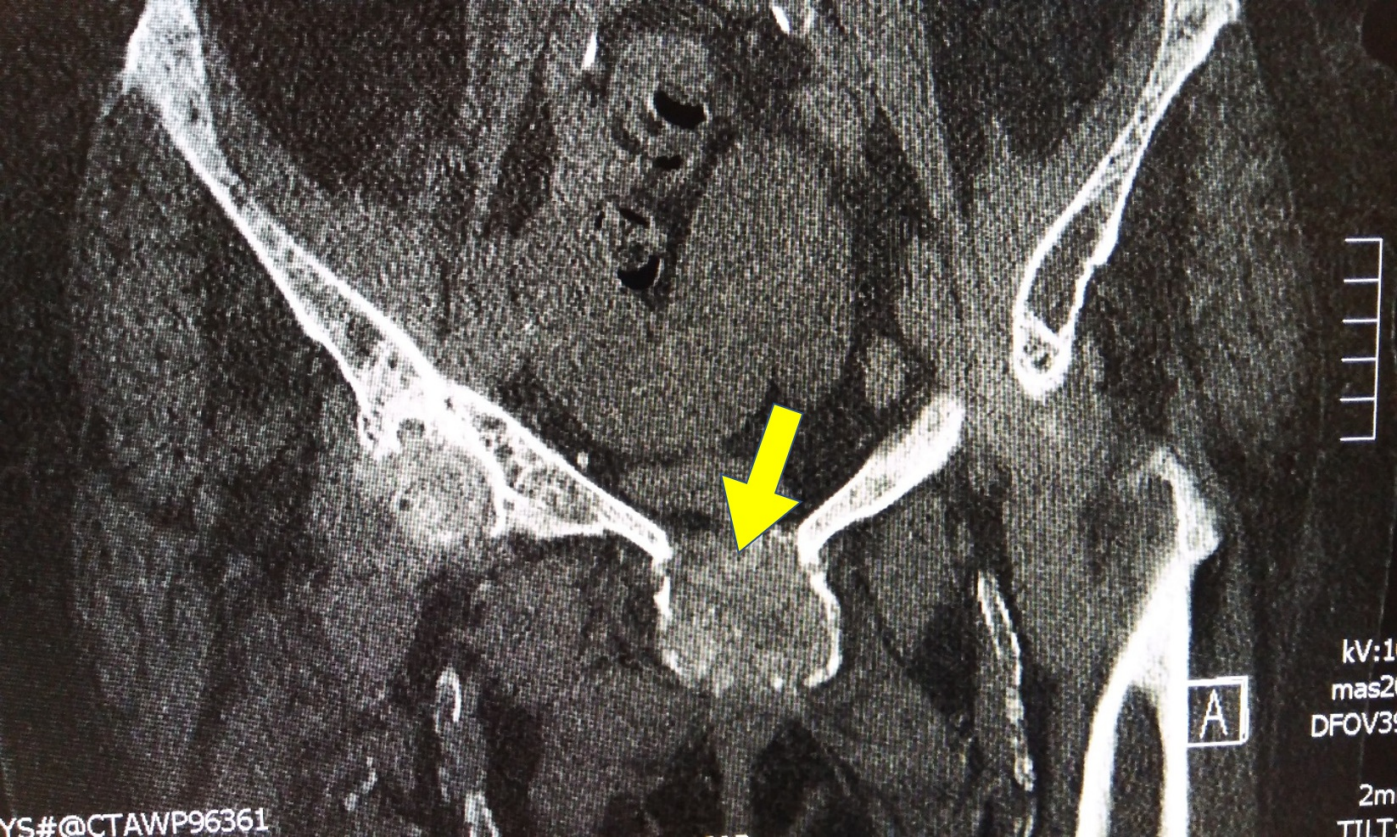
Computed tomography (CT) scan of the pelvis - axial view Chronic pubic symphysitis in the axial view.

Chronic symphysitis (visible in Figure 3 and Figure 4 ) was present. It was also present on a previous x-ray three years ago. The extent of chronic symphysitis seen here is rare.

Her medical conditions presented a challenging surgical situation. Endocrinology, renal, and anaesthetic teams were involved to optimize outcomes and to follow the usual practice in our hospital requiring hip fractures operated on within 48 hours of presentation.

She was optimized medically to the anesthetic team’s satisfaction to proceed to surgery. Determining the type of operative procedure presented another challenge. As noted on the x-ray and CT scans, the basicervical neck of the femur fracture is very close to the femoral calcar. The usual surgical procedure in these cases is to insert a dynamic hip screw or short cephalomedullary nail. However, given the quality of the bone tissue and the extent of bone resorption, we decided against these procedures and chose a bipolar hemiarthroplasty.

She recovered from anesthetics and slowly started her physiotherapy and rehabilitation. She was also evaluated by the endocrinology and renal teams to treat her underlying medical conditions.

## Discussion

Primary hyperparathyroidism signs and symptoms are summarized in a colloquial rhyme as “stones, bones, abdominal groans, and psychiatric overtones” [5].The stones in this phrase refer to renal stones; the abdominal groans refer to indigestion, nausea, vomiting, and peptic ulcers. The psychiatric overtones refer to depression, lethargy, and loss of memory. The bones refer to osteitis fibrosa cystica (OFC), osteomalacia, and, rarely, Brown tumor of long bones [6].

The above-mentioned phrase was used in the early 1970s because, at that time, primary hyperparathyroidism was diagnosed in its advanced stages. Today, especially in western countries, this condition is incidentally diagnosed at very early stages thanks to routine biochemical investigations [7-8]. However, rare cases of advanced disease still present where bone structure and other conditions match the patient case presented.

OFC is characterized by bone pains, pathological fractures, and skeletal deformities [7]. Brown tumor is a unifocal or multifocal condition with increased osteoclastic activity, bone resorption, demineralization, and replacement with loose connective tissue [7].

PTH indirectly stimulates osteoclasts by binding to receptors on osteoblasts, inducing RANK-L (receptor activator of nuclear factor kappa β ligand) and M-CSF (macrophage colony-stimulating factor), causing the overstimulation of bone resorption. RANK-L is an important factor for signaling of transcription factors which lead to osteoclast differentiation. M-CSF stimulates the expression of RANK-L [9].

## Conclusions

While advanced symptomatic primary hyperparathyroidism bone disease is rare, it should be considered in the differential diagnosis especially given the biochemical changes in serum calcium and PTH levels.The patients presenting with hyperparathyroidism and skeletal fractures should be treated not only surgically but also be evaluated medically.
